# Human–AI Co-Agency for Competency-Based Talent Development: A Framework Integrating Creativity, Cognitive Skills, and Ethical Collaboration

**DOI:** 10.3390/jintelligence14070144

**Published:** 2026-07-09

**Authors:** Maha Alfaleh

**Affiliations:** Department of Curriculums and Instructional Technologies, College of Humanities and Social Sciences, Northern Border University, Arar 73222, Saudi Arabia; maha.alfaleh@nbu.edu.sa

**Keywords:** artificial intelligence, education, simulation, human–AI co-agency, competency-based learning system

## Abstract

The rapid integration of generative artificial intelligence (GenAI) into education offers significant opportunities for talent development; however, current applications remain predominantly tool-centric and lack grounding in cognitive and intelligence theory. To address this limitation, the present study introduces the Human-AI Co-Competency Framework for Education (HACC-E), a human-centered, mastery-driven model intended to promote the synergistic development of creativity and cognitive intelligence. Drawing on contemporary intelligence research, including executive function theory, creative cognition models, and socio-emotional competence frameworks, HACC-E positions AI as a cognitive amplifier that supports higher-order reasoning, reflective thinking, and adaptive problem-solving, rather than as a replacement for human judgment. Employing a design science research methodology, the framework integrates competency-based progression, AI-augmented formative feedback, and simulation-based experiential learning within a governance structure that ensures human oversight and ethical accountability. The model aligns fluid reasoning, systems thinking, creative ideation, emotional regulation, and collaborative intelligence with structured AI-mediated feedback loops and authentic performance evidence. Analytical validation confirms pedagogical coherence, measurable competency mapping, and mitigation of critical AI-related risks, including automation bias and overreliance. By operationalizing distributed human–AI agency within a mastery-based progression architecture, HACC-E advances a theoretically grounded approach to AI-enabled talent development. This framework contributes to the ongoing discourse on human-centered artificial intelligence by providing a scalable and ethically robust model for cultivating cognitive, creative, and socio-emotional intelligence in educational systems designed for future readiness.

## 1. Introduction

Education systems worldwide are undergoing a profound transformation driven by accelerating technological change, evolving workforce demands, and increasing expectations for accountability and learning transparency. Traditional education models, largely structured around time-bound curricula, content delivery, and summative examinations, have historically been effective in transmitting foundational knowledge but are increasingly criticized for their limited capacity to demonstrate applied competence and higher-order cognitive skills (Amirova, 2025; Bhardwaj et al., 2025). Numerous studies have highlighted that graduates assessed primarily through examinations often struggle to transfer theoretical knowledge into real-world problem-solving contexts, particularly in complex, interdisciplinary, and uncertain environments (Godwin et al., 2025; Rawle et al., 2025; Xu & Wu, 2025). As a result, higher education institutions face mounting pressure from accreditation bodies, employers, and policymakers to shift toward outcome-based and learner-centered models that emphasize demonstrable competencies rather than content completion. Competency-based education (CBE) has emerged in response to these challenges, offering a paradigm that prioritizes mastery, performance evidence, and flexible learning pathways (Evans et al., 2020; Yao & Lin, 2025). Prior research indicates that CBE improves alignment between educational outcomes and professional expectations by explicitly defining competencies and assessing learners through authentic tasks (McMullen et al., 2023; Ponomariovienė et al., 2025). However, despite its conceptual strengths, CBE implementation remains uneven and methodologically fragmented, particularly in large-scale or technology-enhanced learning environments (Chigbu et al., 2023; Maya Ortiz, 2025; Rogers, 2021). Many institutions struggle to operationalize competencies in a manner that is scalable, assessable, and pedagogically coherent, often reverting to traditional assessments that undermine the intent of competency-based approaches (Cano et al., 2023; Lurie & Garrett, 2017; Singun, 2025). This unresolved tension underscores the need for integrative educational frameworks that can systematically support competency development while accommodating emerging technologies and diverse learner trajectories.

The rapid emergence of generative artificial intelligence (GenAI) has further intensified debates about the future of education. GenAI systems demonstrate unprecedented capabilities in generating explanations, feedback, simulations, and creative outputs, positioning them as potentially transformative tools for personalized learning and formative assessment (Liu & Zhong, 2025; Murtaza et al., 2025; Sousa & Cardoso, 2025). Recent literature reports positive outcomes associated with AI-supported adaptive feedback, intelligent tutoring, and learning analytics, particularly in enhancing learner engagement and self-regulated learning (Tan et al., 2025; Yaseen et al., 2025). At the same time, scholars have raised substantial concerns regarding the uncritical adoption of GenAI in educational contexts. These concerns include automation bias, erosion of critical thinking, lack of transparency in algorithmic decision-making, threats to academic integrity, and inequitable outcomes driven by biased training data (Al-Zahrani, 2024; Chinoracky & Stalmasekova, 2025; Marín et al., 2025). Importantly, many existing AI-in-education implementations conceptualize AI as a supplementary tool rather than as a component of a coherent pedagogical and assessment system. This tool-centric perspective often results in fragmented integration, where AI-generated outputs are detached from learning outcomes, competency frameworks, and institutional governance structures (Albasry et al., 2025; Heil et al., 2025).

Furthermore, the delegation of evaluative or instructional authority to AI systems without clear human oversight mechanisms has been widely criticized as pedagogically and ethically problematic. The literature increasingly emphasizes the necessity of human-centered AI models in which educators retain authority over learning objectives, assessment judgments, and ethical decisions (Alfredo et al., 2024; Jesus & Caumeran, 2026; Le Dinh et al., 2025). However, despite growing consensus on these principles, there remains a lack of structured frameworks that operationalize human–AI co-agency in a way that is educationally meaningful, ethically robust, and aligned with competency-based learning.

Experiential and simulation-based learning approaches have long been recognized as effective mechanisms for developing applied skills, professional judgment, and complex problem-solving abilities (Alharbi et al., 2024). Grounded in constructivist and experiential learning theories, simulation-based education enables learners to engage with realistic scenarios, make decisions, observe consequences, and refine their understanding through iterative feedback. Prior research demonstrates that simulations support deeper learning by bridging the gap between abstract theory and practical application, particularly in domains requiring systems thinking, ethical reasoning, and interdisciplinary co-agency (Dai & Ke, 2022). In competency-based contexts, simulations offer a powerful means of generating authentic performance evidence, allowing competencies to be assessed through observable behaviors rather than proxy indicators such as test scores (Chacko, 2017; Fawns et al., 2026). Advances in digital technologies have expanded the scope of simulation modalities, including virtual laboratories, scenario-based learning environments, digital case studies, and role-play simulations. Despite their demonstrated effectiveness, simulations are frequently implemented as isolated instructional interventions rather than as integral components of a broader competency architecture. Moreover, their potential synergy with AI-driven feedback and analytics remains underexplored in the literature. While some studies examine AI-enhanced simulations, few provide a system-level perspective that aligns simulation design, AI augmentation, competency assessment, and ethical governance (Camilleri, 2024; Schicktanz et al., 2023). This fragmentation limits the scalability, consistency, and institutional adoption of experiential learning approaches, reinforcing the need for integrative frameworks that embed simulations within coherent learning and assessment systems.

Recent empirical studies examining the impact of large language models (LLMs) on higher-order cognitive skills provide a detailed and evolving picture (Martínez-Peláez et al., 2025; Peláez-Sánchez et al., 2024). A growing body of evidence suggests that LLMs can support the development of critical thinking, problem-solving, and creative reasoning by enabling interactive dialog, personalized feedback, and structured knowledge synthesis in higher education contexts (Shahzad et al., 2025; Williams, 2025). For instance, systematic reviews indicate that LLM-supported learning environments can enhance active learning and foster applied problem-solving competencies when aligned with pedagogical objectives (Y. Shi et al., 2026). At the same time, emerging empirical research highlights significant risks associated with unstructured use, including cognitive offloading, reduced metacognitive engagement, and overreliance on AI-generated outputs, which may limit the development of higher-order reasoning skills (Galindo-Domínguez et al., 2026). Notably, several studies report that while generative AI tools effectively support lower-order cognitive processes, such as understanding and application, their contribution to advanced skills, such as evaluation, critical analysis, and creative synthesis, remains inconsistent and highly dependent on instructional design (Adejumo et al., 2026). These findings underscore the need for structured, human-centered frameworks that guide the integration of LLMs in ways that preserve epistemic agency and actively cultivate higher-order cognition.

The reviewed literature reveals a critical gap in contemporary education research and practice: while competency-based education, generative AI, and simulation-based learning are each well established, they are rarely integrated within a unified, governance-aware framework that clarifies their interaction, accountability, and ethical boundaries. Existing approaches tend to emphasize individual tools or pedagogical strategies rather than system-level design, resulting in fragmented implementations and unresolved concerns related to assessment validity, learner overreliance on AI, and academic integrity. To address this gap, this study adopts the concept of human–AI co-agency as a central theoretical construct. Within the context of socio-technical systems theory, co-agency is defined as a structured distribution of roles and decision-making capacities between human actors and artificial intelligence systems, in which both entities actively contribute to learning processes while maintaining distinct domains of authority and accountability. Unlike general notions of human–AI collaboration, which often imply loosely coordinated interaction, co-agency emphasizes that agency is deliberately designed, bounded, and governed. In this model, AI functions as a generative and adaptive cognitive partner that supports feedback, reflection, and exploration, whereas humans retain epistemic authority over judgment, validation, ethical decision-making, and learning progression. This distinction ensures that AI augments, rather than substitutes, human intelligence, thereby preserving educational integrity and aligning with socio-technical principles of human-centered system design. The novelty of HACC-E lies in its explicit operationalization of human–AI co-agency, its use of simulations as primary sources of competency evidence, and its alignment with outcome-based accreditation and quality assurance requirements. This study aims to develop and analytically validate the HACC-E framework using a design science research methodology. The specific objectives are to: (i) define a future-ready competency architecture; (ii) formalize the role of generative AI as an augmentative, not substitutive, educational agent; (iii) embed simulation-based learning as a core assessment mechanism; and (iv) establish governance structures that ensure ethical, transparent, and accountable AI-enabled education.

## 2. Theoretical Framework

**Figure 1:**
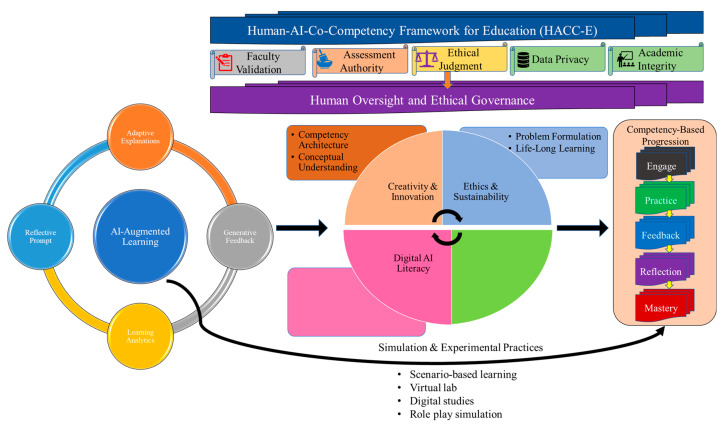
Human–AI competency framework for education (HACC-E).

The Human–AI Co-Competency Framework for Education (HACC-E) is based on the presence of well-founded educational and socio-technical theories that substantiate the design of the framework at the system level as a whole (Figure 1). Figure 1 illustrates the overall architecture of the HACC-E framework, highlighting the interaction between competency development, AI-augmented learning processes, simulation-based environments, and governance mechanisms. The figure emphasizes the systemic integration of these components rather than their isolated function, showing how competencies are developed through iterative cycles of engagement, feedback, and validation within a human-centered AI ecosystem. HACC-E does not implement new principles of pedagogical activity in isolation, but incorporates them into a logical system, combining competency-based learning, constructivist and experiential conceptual inclinations of learning, socio-technical systems theory, and human–AI co-creation models into a unified system to consider both the effectiveness of pedagogical activities and the ethical management (Dastanpour et al., 2017; Wang et al., 2025). Integrating AI in education is aided by such stipulations in this theoretical integration, ensuring that AI-augmented education is learner-centered, measurable, and institutionally accountable.

### 2.1. Competency-Based Learning

Competency-based learning (CBL) or outcome-based education (OBE) is a concept that views education as a cumulative growth and expression of integrated competency as an alternative to graduation of a periodically defined unit of instruction. Unlike the course-centered approaches, CBL focuses on well-defined learning outcomes, a learning progression pegged to mastery, and performance evidence that is manifested in authentic performances (Alani & Grewal, 2024). Authoritative standards like ABET standards of accreditation, the European Qualifications Framework (EQF), and outcome-based higher education models all tend to believe in demonstrable competence, alignment of outcomes and assessment, and transparency of learning progression (Almuhaideb & Saeed, 2020; Hussain Khan, 2019). Empirical and policy-based research indicates that competency-based methods enhance the employability of graduates, the relevance of curriculum, and accountability through directly connecting learning processes with workplace demands.

In the framework of HACC-E, competency-based learning serves as the framework’s structural backbone, and it orchestrates curriculum design, learning activities, assessment mechanisms, and follows the logic of progression. The competencies are established as synthesized skills that include conceptual knowledge, applied problem-solving, ethical decision-making, digital and Artificial Intelligence literacy, teamwork, and lifelong learning. Promotions depend on acquired expertise as opposed to time spent in classrooms, which permits adaptable and individualized learning routes.

Table 1 summarizes a theoretical justification of the HACC-E framework. Comparing the traditional modes of education with the HACC-E model, based on dimensions such as curriculum design, attention to assessment, teacher role, and learning presence, Table 1 reveals structural discrepancies that persist in the fields of conventional education. Such inconsistencies, especially the reliance on indirect evaluation and temporal flow, are not in line with the logic of accreditation as implied in the ABET, EQF, and OBE models. HACC-E is a response to such conflicts in repositioning competencies as the key educational design and assessment unit, therefore, harmonizing institutional practice with the set standards of quality assurance and accreditation.

### 2.2. Constructivist and Experiential Learning

The constructivist theory of learning assumes that learners construct knowledge by participating, reflecting on, and interacting with their surroundings instead of having information imposed on them. Experiential learning goes further to apply this principle through the focus on iterative action, feedback, reflection, and refinement cycles. Education research indicates that students will gain a deeper insight and marketable skills when involved in real-life exercises resembling a complex aspect of life (Hvalby et al., 2024; H. Shi et al., 2024). Such principles especially apply to competency-based education in which learning outcomes should be observable, context-effective, and applicable.

**Figure 2:**
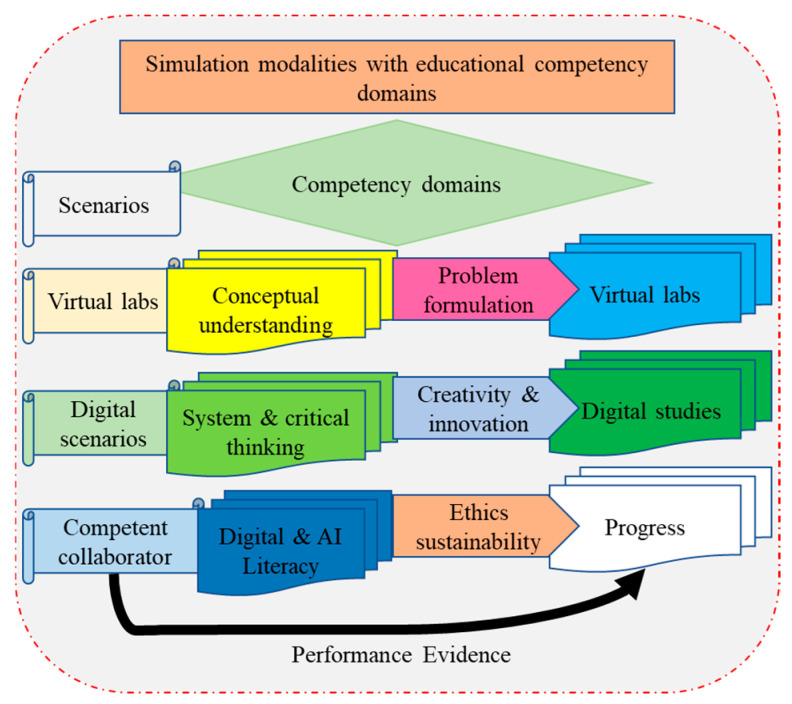
Alignment of simulation modalities with educational competency domains.

Simulation-based learning is a fully developed form of experiential learning theory in which students can interact with real-life conditions, put their choices into practice, and see the outcomes of various situations under conditions of controlled learning environments. The concept of simulations has two applications in competency-based settings: simulations can be used both as a learning platform and as an evaluation tool that can produce realistic performance data (Kasperski et al., 2025; Munim et al., 2025). Contrary to the classical examinations, simulations are used to learn how to make more decisions, how to solve a problem, how to cooperate in group work, how to think about the ethical issues, and so on, all the aspects that lie at the heart of integrated competencies development. The experience in which HACC-E directly incorporates simulation-based experiential learning into its competence structure is to ensure that the abstract learning outcomes are translated into observable behaviors. The alignment is shown conceptually in Figure 2, which shows how various simulation modalities (including scenario-based learning, virtual laboratories, digital case studies, and role-play simulations) operationalize each of the domains of educational competencies. Figure 2 presents the competency architecture underlying the HACC-E framework. It categorizes competencies into cognitive, creative, socio-emotional, and digital domains, while also illustrating their interdependencies. The figure highlights that these competencies are not developed in isolation but emerge through integrated learning processes supported by AI and experiential engagement.

The fact that simulation outputs are used as performance evidence, which is further evidenced in Figure 2, supports the validity of competency assessment. HACC-E addresses a weakness that is prevalent in the literature because, in most cases, experiential learning is discussed as an add-on activity aimed at developing one specific aspect of a curriculum or learning process instead of being a central element of an outcome-based evaluation framework (Asim et al., 2021).

### 2.3. Socio-Technical Systems Theory

The socio-technical systems theory creates an image of organizations as complex systems whereby results are reached because of interactions between social actors, technologies, institutional structures, and governance mechanisms. This view is used in the learning field to oppose tool-based perspectives of innovation, whereby learning outcomes are made by not only instructional technologies, but also human roles, policies, assessment practices, and ethical standards. The emergence of AI as an educational technology is an indicator of the topicality of the given point of view since AI systems no longer contribute to learning but are active participants in the process (Laupichler et al., 2022). HACC-E takes an approach of socio-technical framing by specifically describing education as an interrelated system of learners, educators, AI systems, simulations, competencies, and governance systems. Such an approach will justify why AI governance cannot be regarded as a layer of policies. Absent explicit regulation, AI-aided learning is likely to exacerbate inequality, undermine the validity of assessments, and devalue educational degrees. HACC-E can therefore be explained by socio-technical theory as the layered architecture ensures that the pedagogical processes are never considered outside the ethical supervision, the data management, and the accountability of the institution (Gibreel & Hong, 2017; Navarro-Bringas et al., 2020). The way HACC-E integrates governance into the system, as opposed to treating it as an outlier issue, allows us to tie the concepts of technological innovation with the values of education and social responsibility.

### 2.4. Human–AI Co-Creation and Distributed Agency

In this study, the concept of human–AI co-agency extends beyond conventional interpretations of human–AI collaboration by explicitly framing agency as a distributed and governed property of the socio-technical system. Co-agency refers to the intentional allocation of cognitive, operational, and evaluative roles between humans and AI systems, such that both participate in the learning process without blurring accountability boundaries. While collaboration typically denotes cooperative interaction, co-agency introduces a structural dimension in which responsibilities are differentiated: AI contributes generative capabilities, adaptive feedback, and analytical support, whereas human actors retain authority over interpretation, validation, ethical reasoning, and final decision-making. This distinction is critical in educational contexts, where unstructured delegation to AI may lead to automation bias, erosion of critical thinking, and compromised assessment validity.

**Figure 3:**
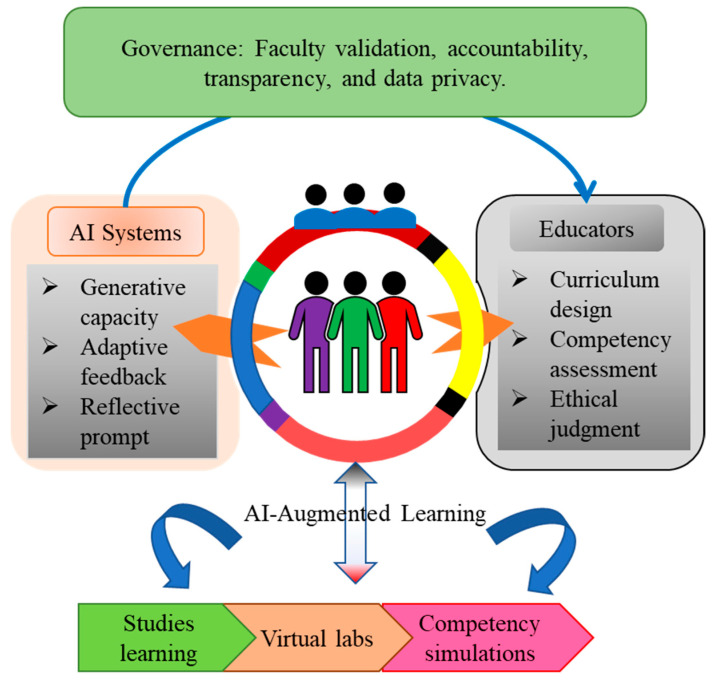
Distributed human–AI agency model in education.

The human–AI co-creation theory focuses on joint interaction between human and AI in which agency is not given to automation but is distributed. The former is vital in an educational environment to prevent automation bias, the tendency to excessively rely on the outputs of AI at the peril of human judgment. Studies always caution against an uncritical use of AI, as it may lead to a reduction in critical thinking in learners and also lead to a loss of professional authority on the part of teachers. The way HACC-E implements distributed agency is clearly revealed in the distribution of human and AI functions, which are explicitly shown in Figure 3. Figure 3 depicts the learning and assessment cycle within HACC-E, emphasizing the role of simulation-based environments and AI-augmented feedback. The cyclical structure reflects the iterative nature of competency development, where learners continuously engage in task execution, receive feedback, reflect on performance, and refine their understanding under human supervision. According to this model, AI will be used as a source of generative capacity, open-loop use of reflection, and teaching feedback, allowing the educator to make more decisions regarding the curriculum, validating assessments, ethical decision-making, and progression decisions. The learners interact with AI as a cognitive amplifier and not as an evaluator of authority. Such a transparent division of the agency maintains teacher authority and institutional responsibility and utilizes the scalability and personalization opportunities of AI. HACC-E reduces the risks of automation bias and academic dishonesty by introducing human control into the process of learning and assessment. Figure 3 is therefore not only a conceptual example, but a governing system to understand the scope of accountability and to strengthen the human focus of the framework.

To enhance conceptual clarity, Table 2 synthesizes the relationships between the key theoretical foundations underpinning the HACC-E framework. Rather than functioning as isolated perspectives, these theories are integrated to collectively inform the design of AI-augmented, competency-based learning environments.

This synthesis illustrates that HACC-E is not a simple aggregation of theories but a coherent integration in which constructivist pedagogy defines how learning occurs, socio-technical systems theory defines how human and AI elements interact, and co-agency operationalizes this interaction within competency-based and simulation-driven environments.

## 3. Methodology

To build and analytically justify the Human–AI Co-Competency Framework for Education (HACC-E), this research paper takes a design science research (DSR) approach. DSR is especially applicable to research aimed at solving complex real-world challenges by establishing rigorously based artifacts, e.g., a model, framework, or architecture, as opposed to only the testing of hypotheses. Since generative AI in the field of education is emerging and changing at a very rapid pace, a design-based methodological approach permits the integration of theory and the development of frameworks, as well as the systematic validation and justification of their validity without premature and context-related empirical assertions.

### 3.1. Design Science Research Justification

The research of design science is very common in the field of education, information systems, and socio-technical research in cases where there is a need to develop a prescriptive artifact that enriches practice and cognition in a problem. As opposed to an explanatory or predictive research paradigm, DSR focuses on formulating, refining, and validating the facts to fill gaps that have been identified between existing theory and practice. The artifact in the current research is not a software system or an instructional intervention but a conceptual educational framework, and, therefore, DSR would be an apt methodologically recognized option.

The key educational challenge that was covered in this study is the poor and unregulated unification of competency-based learning, generative artificial intelligence, and simulation training. The current literature offers little guidance on how these elements are to interact with one another, as it is more likely to treat them separately. DSR facilitates the aggregation of all these components into one comprehensive framework based on theory and in line with the requirements of institutional quality assurance.

An empirical experiment is not the sole method used to validate in design science, especially concerning conceptual artifacts. Rather, validity is determined by theoretical endorsement, internal consistency, logical consistency, and adherence to prevailing criteria and standards. This research, therefore, takes the form of analytical validation with appropriate results and not empirical validation. The quality of the HACC-E framework is then evaluated in terms of being theoretically sound, pedagogically sound, measurable, morally sound, and applicable in real-life educational scenarios. This strategy is also in line with conventional DSR guidelines, which recognize that early-stage artifacts or system-level evidence are broadly acknowledged in the peer-reviewed literature.

### 3.2. Artifact Design and Iteration

The HACC-E framework was designed on an iterative and principle-based design approach. As opposed to being a product of incremental conceptualization, the framework was developed through the process of feedback loop expansion that considered legal mandates and education-based theories of knowledge and ethical concerns about AI implementation.

The development of the artifact was based on the following four design principles:

**Primacy of Competencies**: Competencies should serve as the overall organizing principle of curriculum design, learning activities, assessment, and progression. This principle informed the competency architecture in Section 4 and is the basis of the setup of the framework, as shown in Figure 1.

**Human-centered AI Augmentation**: AI systems are not supposed to make human judgment secondary during learning and assessment. This direction was the direct guide in creating the model of distributed human–AI agency presented in Figure 3, where the control of goals, evaluation, and ethics is in human possession.

**Authentic Performance Evidence**: Competency achievement should be exhibited in the form of observable performance that is context-rich as opposed to indirect proxies. This concept informed the design of the combination of simulation-based experiential learning and the direct correlation between simulation modalities and domains of competencies demonstrated in Figure 2.

**Governance by Design**: The ethical oversight, data protection, and academic integrity have to be integrated into the framework instead of being off-putting policies. This principle informed the incorporation of governance as one of the core framework layers and not as an augmentation factor.

The framework architecture was further graphically developed to provide consistency among these principles, and it achieved the layered system model as shown in Figure 1 and the adaptive learner progression workflow as shown in Figure 2.

### 3.3. Strategy of Analytical Validation

To establish the rigor and credibility of the HACC-E framework, an analytical validation strategy was employed. This approach evaluates the conceptual artifact against four widely accepted criteria in educational and socio-technical systems: pedagogical coherence, assessability, ethical robustness, and scalability.

Assessability was evaluated by analyzing whether the framework enables the generation of valid and observable evidence across competency domains. The use of simulation outputs such as decision pathways, strategies applied, and outcomes achieved addresses a common limitation of traditional assessment models. The explicit mapping between competencies and simulation modalities in Figure 2 supports transparent and defensible assessment practices aligned with outcome-based accreditation requirements.

Ethical robustness was examined through the framework’s capacity to mitigate known risks associated with generative AI, including automation bias, overreliance on AI-generated outputs, and threats to academic integrity. The distributed human–AI agency model presented in Figure 3, together with embedded governance structures, ensures that ethical responsibility and accountability remain human-led rather than algorithmically delegated.

**Figure 4:**
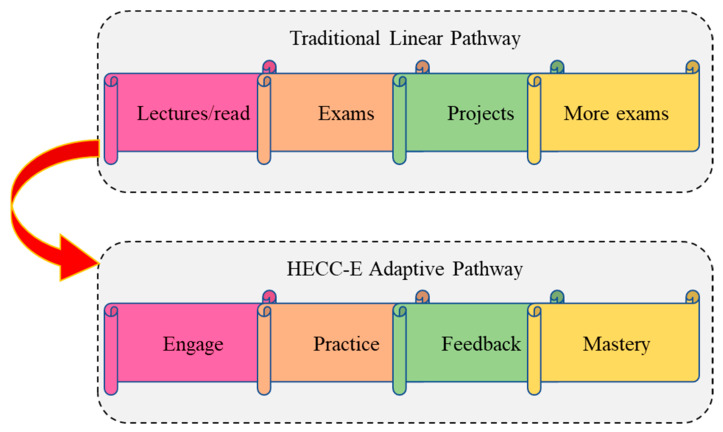
Comparison of traditional linear pathway and HECC-E adaptive pathway.

Scalability was assessed by evaluating whether the framework can be adapted across disciplines, institutional contexts, and diverse learner populations. By decoupling progression from instructional time and embedding AI-supported feedback mechanisms, HACC-E supports flexible and potentially scalable implementation. The adaptive progression logic illustrated in Figure 4 further demonstrates how mastery-based pathways can replace traditional linear models. Taken together, these analytical dimensions indicate that HACC-E constitutes a theoretically coherent and practically relevant framework. While the present validation is conceptual in nature, the structure of the framework supports future empirical testing and application across varied educational settings.

### 3.4. Analytical Validation Strategy

To establish the rigor and credibility of the HACC-E framework, an analytical validation strategy was employed. This strategy evaluates the artifact against four widely accepted criteria for educational and socio-technical systems: pedagogical coherence, accessibility, ethical robustness, and scalability.

Pedagogical coherence was assessed by examining the alignment between learning theories, competency definitions, AI-augmented processes, and simulation-based activities. The framework demonstrates coherence by ensuring that competencies drive learning design, AI functions support formative learning, and simulations provide authentic contexts for application. The consistency of this alignment is visually synthesized in Figure 1 and Figure 2.

Accessibility was evaluated by analyzing whether the framework enables the generation of valid and observable evidence for each competency domain. The use of simulation outputs such as decision pathways, strategies applied, and outcomes achieved addresses a common weakness in traditional assessment models. The explicit mapping between competencies and simulation modalities in Figure 2 supports transparent and defensible assessment practices aligned with outcome-based accreditation requirements.

Ethical robustness was examined through the framework’s capacity to mitigate known risks associated with generative AI, including automation bias, overreliance on AI-generated outputs, and threats to academic integrity. The distributed agency model in Figure 3 and the embedded governance structures ensure that ethical responsibility and accountability remain human-led rather than algorithmically delegated.

Scalability was assessed by evaluating whether the framework can be adapted across disciplines, institutional contexts, and learner populations. By decoupling progression from instructional time and embedding AI-supported feedback mechanisms, HACC-E enables flexible, scalable implementation without compromising assessment integrity. 

Together, these analytical validation dimensions demonstrate that HACC-E constitutes a theoretically sound and practically viable educational framework, suitable for future empirical evaluation and institutional adoption.

## 4. Human–AI Co-Competency Framework for Education (HACC-E)

### 4.1. Framework Overview

The conceptualization of education proposed by the Human–AI Co-Competency Framework for Education (HACC-E) is that of a human-focused, AI-enhanced, competency-based socio-technical system. As opposed to tuning technology, pedagogy, or assessment to form distinct levels of a framework, it incorporates all of them in an active network aimed at enabling real learning, valid assessment, and ethical accountability. The general framework and dependencies are shown in Figure 1. Figure 1 includes visual layers that are a deliberate and conceptual design. The competency architecture will serve as the base layer because competencies will be the main educational outputs and the unit of organization in the curriculum structure and evaluation. AI-enhanced learning processes (operating upward) support such competencies through adaptive feedback, scaffolding, and analytics as opposed to stipulating results. The next level is simulation-based experiential practice, which converts abstract competencies into performance by way of real-world tasks that involve participating in authentic and context-laden simulations. Lastly, the system has human control and ethics that cover the whole system, which makes it strongly humanistic in terms of authority on pedagogical, assessment, and ethical responsibility.

HACC-E has neither a linear flow of learning nor a cyclic learning process but rather an adaptive cycle. Competency-based engagement serves as a starting point through which learning proceeds by AI-powered formative feedback and simulated application, and ultimately through a demonstration of acquired knowledge as performance artifacts. Governance operates on this cycle, not at its end, so that the cycles of validation and accountability are maintained continuously. Most notably, governance is seen as a facilitating layer but not as a limiting factor: it is a way to legitimize AI applications and ensure the agency of the learners and institutional credibility. Integration at multiple levels is a distinguishing feature that sets HACC-E apart from tool-focused or modular methods and positions it as a holistic framework of AI-assisted competency-based learning.

### 4.2. Competency Architecture

The fundamental belief in HACC-E is in a forward-looking competency architecture, which is expected to remain pertinent in the face of technological, professional, and social transformation. The competency areas that are found in Table 3 are not just limited to technical skills but also include cognitive, ethical, and interpersonal skills that are integrated. These areas, such as conceptual understanding, problem development, systems thinking, critical thinking, creativity, digital and AI literacy, ethics and sustainability, communication, and lifelong learning, are relevant to competencies that have always played a significant role in modern accreditation systems and analyses of the future workforce.

To enhance the operational clarity of the competency architecture, selected competencies are further illustrated through representative sub-indicators and pedagogical scenarios. These examples demonstrate how abstract competency domains manifest as observable learner behaviors in AI-augmented and simulation-based learning environments (Table 4).

This architecture will be future-proof due to its orientation on capabilities. Instead of focusing on competencies related to certain devices, platforms, or technologies, HACC-E also focuses on transferable skills that remain intact despite changes in technology. This difference is especially important in relation to AI literacy, which is more accurately defined as the ability to respond to AI products critically, understand constraints and biases, exercise sound judgment, and act ethically when interacting with AI. Considering AI literacy as the use of a tool may create the risk of obsolescence and surface learning quickly; viewing it as a meta-competency makes it flexible and responsible. Arranging learning in these integrated areas, through HACC-E, facilitates clarity in matching the curriculum design, learning activities, and evaluation evidence. Table 2 is thus not primarily a list of results, but a design scaffold that gives information to all the further strata of the structure.

In addition to cognitive and technical competencies, the socio-emotional dimension of HACC-E encompasses learners’ capacity for emotional regulation, resilience, confidence, and adaptive engagement in complex learning environments. Within this domain, academic anxiety emerges as a critical factor influencing learner performance, motivation, and persistence, particularly in AI-augmented educational contexts.

Recent research suggests that generative AI systems can both mitigate and exacerbate academic anxiety, depending on how they are integrated into learning processes. On the one hand, AI-supported environments may reduce anxiety by providing immediate, non-judgmental feedback, enabling learners to explore ideas iteratively without fear of evaluation, and supporting self-paced learning. This can enhance learner confidence, especially for those who may hesitate to engage in traditional classroom interactions.

On the other hand, unstructured or opaque use of AI may intensify anxiety by fostering overreliance, reducing learners’ sense of competence, and creating uncertainty regarding the validity of their own knowledge. Learners may become dependent on AI-generated responses, leading to diminished self-efficacy and increased cognitive dissonance when required to perform independently. Additionally, concerns about academic integrity, evaluation fairness, and the perceived “black-box” nature of AI systems may further contribute to stress and disengagement. HACC-E addresses these challenges by embedding socio-emotional support within its human–AI co-agency model. Through structured reflection, guided AI interaction, and human-in-the-loop validation, learners are encouraged to critically engage with AI outputs rather than passively accept them. Simulation-based experiential learning further supports confidence-building by allowing learners to practice decision-making in low-risk environments, while human oversight ensures that feedback and assessment processes remain transparent and supportive. In this way, HACC-E positions AI not only as a cognitive amplifier but also as a regulated component of a psychologically supportive learning ecosystem.

### 4.3. AI-Augmented Learning Processes

**Figure 5:**
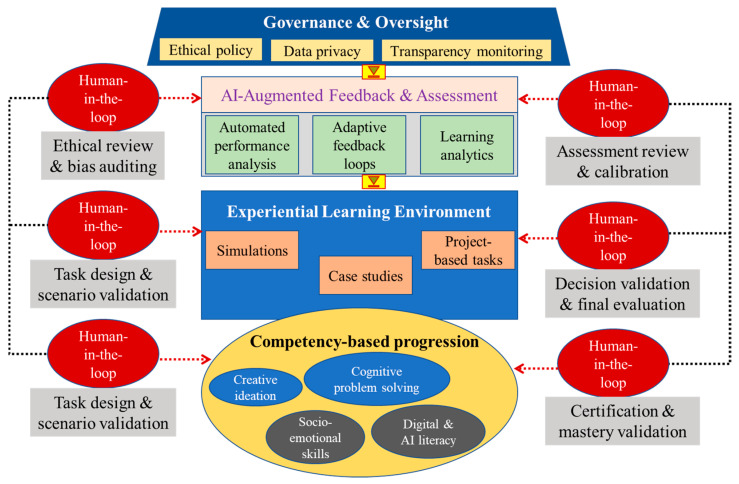
Learner progression workflow in the HACC-E framework.

AI-enhanced learning in HACC-E is also clearly created to facilitate formative learning and reflective performance, instead of automating teaching and evaluation (Figure 5). The evolution of the learner involves several steps, which are described by the learner’s progression workflow (Figure 5) and turn this piece of art into a testimony of pedagogical reasoning, as opposed to visual embellishment.

It starts with the involvement of the learners in competency-based tasks placed in real-world settings. At this level, students proactively formulate issues, learn, and develop preliminary plans. The generative AI then provides adaptive formative feedback, such as explanations, views on alternative perspectives, reflection questions, and suggestions for improvement. Notably, such AI contributions are contextual and restricted to set competency objectives and are not completely produced out of thin air.

This response triggers a process of self-reflection, in which students critically assess AI outputs, subject those outputs to confirmation with the knowledge of the domain, and polish their own knowledge as a result of justifiable inquiries. This process is monitored by teachers whose role is limited to being mentors and validators of the content, and not deliverers of the content. This cycle ends with the demonstration of competency in which learners put to use the polished knowledge in simulation-based activities that produce performance evidence. Incorporating AI into this organized process, Figure 5 shows how HACC-E can use AI to its full advantage without abusing learner agency or teacher control.

To further operationalize the governance structure, the HACC-E framework explicitly incorporates multiple human-in-the-loop intervention points across the AI-augmented learning and assessment cycle. These intervention points ensure that automated processes remain subject to pedagogical judgment, ethical accountability, and institutional standards.

First, human educators intervene at the task design and competency alignment stage, where learning objectives, assessment criteria, and simulation parameters are defined. This ensures that AI-generated feedback and learning pathways remain aligned with intended competency outcomes. Second, during the AI-generated formative feedback phase, instructors perform periodic validation of AI outputs, particularly in high-stakes or conceptually complex tasks. This includes reviewing explanations, identifying potential inaccuracies or bias, and guiding learners in critically evaluating AI-generated suggestions.

Third, a critical human-in-the-loop checkpoint occurs at the assessment and performance evaluation stage, where simulation outputs and learner artifacts are reviewed and validated by educators. Rather than relying on automated scoring, HACC-E adopts a hybrid evaluation model in which AI supports data aggregation and pattern recognition, while human assessors retain authority over judgment, grading, and competency verification. Fourth, human oversight is embedded in the ethical governance layer, including bias auditing, academic integrity monitoring, and data governance. Educators and institutional stakeholders are responsible for identifying anomalies, ensuring fairness, and enforcing accountability standards in AI-mediated processes. These explicitly defined intervention points, illustrated in Figure 5, transform governance from an abstract principle into a structured mechanism of control. By embedding human judgment at critical stages of task design, feedback validation, and assessment, HACC-E ensures that AI functions as an augmentative system while preserving the integrity, fairness, and accountability of educational outcomes.

### 4.4. Simulation-Based Experiential Practice

The main way in which HACC-E converts learning into assessable competence is simulation-based experiential practice. The simulations offer true-to-life circumstances where the learners deal with the circumstances of complexity, uncertainty, and ethical trade-offs that are a reflection of the real-life professional environment. HACC-E regards simulations as core assessment tools that generate direct performance evidence; they are not an additional activity.

Figure 2 explicitly shows the coordination of the simulation modalities against the competency domains. This number illustrates how virtual laboratories, systems thinking, ethical reasoning, creativity, and co-agency are the competencies that are operationalized by means of scenario-based learning, virtual laboratories, digital case studies, and role-play simulators. HACC-E is also capable of doing this by mapping the outcomes of simulations against individual competencies, a common problem in the research of experiential learning that assumes that learning gains have taken place, rather than having been proven.

Accreditation-wise, this approach helps quality assurance to be based on outcomes as it enables the production of assessment artifacts that are transparent, observable, and defensible. The results of simulations, including decision paths, used strategies, and results analysis, allow the educators to measure the accomplishment of competencies with fixed standards, enhancing not only the academic rigor but also the institutional accountability.

### 4.5. Human Oversight and Ethical Governance

Human supervision and moral management do not bear any negotiation when it comes to the HACC-E framework. Instead of defining ethics as a regulatory framework in pedagogy, HACC-E considers governance an integral part of the learning system. The main ethical risks related to the implementation of generative AI and related mitigation measures are listed in Table 5, which should be regarded as a risk governance matrix and not a descriptive checklist.

Table 5 illustrates the efforts of mitigating the effects of risks like bias of the algorithm, dependence on AI, assessment invalidity, data breach, and breach of academic integrity with explicit human verification, clear assessment procedures, and an institutional governance framework. Such framing explains why full automated evaluation is clearly opposed in HACC-E, but having potential in automated assessment omits the contextual judgment and ethical discernment behind sound competency assessment and may entrust academic judgment to algorithms of dubious complexity. 

Holding human control in each step, such as curriculum development to assessment validation, HACC-E saves the power of educators and the safety of learner agency, as well as accountability. In this way, governance can be regarded as an enabling mechanism to drive AI integration on the one hand and to protect the values of education on the other, contributing to the human-oriented direction of the framework.

## 5. Learning Progression Models

Learning progression models identify the way learners move through the learning programs and also how competency is identified. Linear models of progression are traditionally used in traditional education systems, where students progress through sequential and pre-established sets of courses, which follow a time-based system, characterized by the number of credits and the final examinations. Although this model offers administrative simplicity and standardization, it is increasingly out of step with the emergence of complex, higher-order competencies, including the ability to think in systems, engage in ethical reasoning, be creative, and solve problems adaptively. These competencies are not built consistently or predictably in a learner and also cannot be accurately measured at specific points in time. As a result, linear progression models tend to confound the exposure to instruction and learning achievement so that the knowledge about the real competency achievement becomes less apparent.

This restriction of linear progress is especially noticeable in the learning setting with greater technological capabilities. Students are entering the programs with a range of prior knowledge, learning environments, and cognitive strategies, but linear models are imposing the same pace and test structure. This inflexibility discourages reflective learning, limits individualization, and often rewards superficial learning strategies that focus on examinations and not the acquisition of transferable skills. Furthermore, linear models cannot adequately support AI-augmented learning because they cannot take advantage of the benefits of the AI-based system to offer adaptive feedback and personalized learning experiences. Most AI tools in such systems are usually minimized as ancillary assistants and not as learning propellers.

The HACC-E model develops a progressive, mastery-based, adaptive model that essentially redefines the manner in which learning improvement is established. Figure 4 provides a direct demonstration of this paradigm shift in that the conventional linear pathways are opposed to the possibilities that the adaptive progression that HACC-E provides. In this model, the next level or grade of promotion is dependent on the mastery of the competency demonstrated and not the amount of time taken in instruction. Learners can go through sequences of repetitive learning, feedback, reflection, and implementation till performance can prove a set of predetermined competency concepts. This setup serves both personal learning patterns and hell-bent achievement norms.

While the HACC-E framework is designed as a generalizable and discipline-agnostic model, its implementation can be meaningfully adapted to discipline-specific contexts through the customization of competency emphasis, simulation modalities, and assessment strategies. The core competency architecture remains stable across domains; however, the manifestation of these competencies varies according to disciplinary epistemologies, professional practices, and learning objectives.

For example, in engineering and STEM disciplines, competencies such as problem formulation, systems thinking, and technical reasoning may be operationalized through simulation-based laboratories, computational modeling tasks, and design optimization scenarios, where AI supports data analysis and iterative solution refinement. In contrast, in social sciences and policy studies, competencies may be expressed through scenario-based simulations involving socio-political decision-making, ethical trade-offs, and stakeholder analysis, with AI facilitating access to diverse perspectives and policy modeling. In humanities and research-oriented disciplines, competencies such as critical thinking, creativity, and communication may be demonstrated through AI-assisted research writing, argument construction, and interpretive analysis, where learners critically evaluate AI-generated content and refine their own scholarly voice.

Importantly, these adaptations do not alter the underlying principles of human–AI co-agency, mastery-based progression, and simulation-based assessment. Rather, they illustrate how HACC-E provides a flexible structural framework within which discipline-specific pedagogies can be embedded. This adaptability enhances the framework’s scalability and relevance across diverse educational contexts while maintaining consistency in competency validation and ethical governance.

The personalization made with the help of AI naturally goes along with mastery-driven progression. In HACC-E, generative AI is used to discover learning gaps in individual learners, offer them focused formative feedback, and recommend adaptive learning materials. Nonetheless, decision-making is a human endeavor, so personalization does not compromise evaluation integrity. Figure 4 shows the interaction between AI-enhanced feedback mechanisms and simulation-based performance evidence to create the ability to make flexible but responsible progression paths. HACC-E allows personalization to be scaled without loss of rigor by debriefing progress over time and by including AI in competency validation processes.

This model of adaptive progression is a paradigm shift in which content coverage is supplanted with competency demonstration, even pacing with individual mastery, and valid assessment with ongoing validation. By connecting the logic of progression with the concept of competency-based education and the support of learning analytics with AI, HACC-E represents an alternative to linear educational progressions, which is better aligned to devising a multi-faceted skill set and lifelong learning competencies.

### 5.1. Implementation Guidelines and Use Case Scenarios

To enhance the reproducibility and practical applicability of the HACC-E framework, this section outlines a structured set of implementation guidelines alongside illustrative use case scenarios. These guidelines translate the conceptual architecture into actionable steps for educators and institutions seeking to adopt AI-augmented, competency-based learning systems.

#### 5.1.1. Implementation Guidelines

The implementation of HACC-E can be structured into four sequential phases:

**Competency Mapping and Curriculum Alignment**

Educators first define discipline-specific competencies aligned with the core domains of HACC-E. Learning outcomes, assessment criteria, and performance indicators are explicitly mapped to ensure clarity and measurability.

**Design of AI-Augmented Learning Activities**

Learning tasks are developed to incorporate generative AI as a formative support tool. AI is used to provide explanations, generate alternative perspectives, and scaffold learner reflection, while ensuring that tasks require active human interpretation and decision-making.

**Integration of Simulation-Based Assessment**

Simulation environments are designed to translate competencies into observable performance. These may include scenario-based decision-making tasks, virtual laboratories, role-play simulations, or case-based problem-solving activities.

**Human-in-the-Loop Governance and Validation**

Human oversight is embedded at critical stages, including task design, AI output validation, and final assessment. Educators review AI-generated feedback, evaluate learner performance, and ensure ethical compliance and fairness.

#### 5.1.2. Illustrative Use Case Scenarios

To demonstrate the operationalization of HACC-E, the following examples illustrate its application across different educational contexts:

**Engineering Education Scenario**

Students engage in a simulation-based design project where they develop and optimize a system (e.g., energy-efficient infrastructure). AI supports data analysis and design suggestions, while learners iteratively refine solutions. Educators validate final designs based on performance criteria and system constraints.

**Social Sciences Scenario**

Learners participate in a policy simulation involving complex socio-economic trade-offs. AI provides contextual data and alternative viewpoints, while students analyze implications and justify decisions. Human evaluators assess reasoning quality, ethical considerations, and evidence use.

**Research Writing Scenario**

Students use AI tools to explore literature, generate initial drafts, and refine arguments. They are required to critically evaluate AI-generated content, identify inaccuracies, and develop independent scholarly arguments. Assessment focuses on originality, critical analysis, and argument coherence.

These scenarios illustrate how HACC-E supports the development and assessment of higher-order competencies while maintaining human oversight and ethical accountability. By providing structured guidance and contextual examples, the framework becomes reproducible across diverse institutional and disciplinary settings.

## 6. Discussion

### 6.1. Theoretical Contribution

The research relocates the concept of generative AI in a human-centered, competency-based socio-technical process, instead of positioning it as an instructional or assessment alternative. The HACC-E system adds to the body of knowledge because it explicitly incorporates competency-based education, constructivism and experiential learning, socio-technical systems theory, and co-creation between humans and AI as the unified conceptual framework. In contrast to the current conceptualizations, which focus on these dimensions independently, HACC-E shows how learning outcomes, AI augmentation, experiential practice, and governance have to work as devoid of each other components of the system. This integration explains why AI serves as an integrating element that helps build and reflect on knowledge without thrusting itself into epistemically privileged territory. The framework will resolve the longstanding theoretical issues surrounding AI-mediated settings, such as the automation bias problem and the erosion of professional judgment due to operationalization of distributed agency through defined human and AI roles. In theory, HACC-E applies a human–AI interaction model to the educational sphere because it demonstrates that agency can be distributed without compromising accountability. This is especially important in the context of growing demands to adopt AI that are now theory-aware in education and that shift the paradigm towards principled system construction rather than descriptive or tool-focused ones.

### 6.2. Pedagogical Contribution

On a pedagogical level, HACC-E rehashes learning as the process of engagement, feedback, reflection, and performance, reinforced with AI and confirmed with the help of verifiable evidence. The model adds a systematic approach to the application of AI in pedagogy without compromising deep learning and the agency of the learners. The foundation of HACC-E on generative AI integration into competency-related tasks and reflection loops helps to reduce the risk of passive consumption and shallow interaction that have been most commonly reported in AI-driven learning settings. Use of simulation-based experiential practice as the main source of assessment information makes pedagogical rigor even stronger in that learning activities are in line with the complexity of the real world. This addresses an enduring issue in education, which is measuring higher-order skills such as systems thinking, ethical decision-making, and creativity. HACC-E is designed to support statistically significant observable performance in context-rich settings, instead of using indirect proxies, as is the case with HACC-E. The framework enables pedagogically tailored learning sequences to offer aligned standards and a good opportunity to create homogeneous, high-quality competency training in AI-enhanced learning environments on a large scale.

While the HACC-E framework is designed with scalability and institutional applicability in mind, these outcomes should be interpreted as potential rather than guaranteed. The successful implementation of the framework depends on several contextual factors, including technological infrastructure, institutional readiness, faculty expertise, and governance capacity.

In particular, the integration of AI-augmented learning and simulation-based assessment requires not only access to appropriate digital tools but also the development of pedagogical competencies among educators to effectively design and oversee human–AI co-agency processes. Additionally, institutional policies related to assessment, academic integrity, and data governance play a critical role in shaping how the framework can be operationalized at scale. Therefore, while HACC-E provides a structured and adaptable model that may support scalable transformation in educational systems, its effectiveness and generalizability require systematic empirical validation across diverse institutional contexts. Future research should examine implementation outcomes in real-world settings to assess scalability, sustainability, and impact on learning and assessment practices.

### 6.3. Institutional and Accreditation Relevance

On the institutional level, HACC-E is directly responsive to the growing calls in respect to transparency, accountability, and outcome-based quality assurance. Standards like ABET and the European Qualifications Framework focus on learners showing actual outcomes of learning, their correspondence to testing and competencies, and the documentation of their success. HACC-E gives institutions a system to work through the requirements in AI-enabled environments. The mastery-based logic of progression in the framework, alongside the performance evidence in the form of simulations, promotes defensible assessment practices and decreases the importance of time-based or exam-driven evaluation procedures. In addition, HACC-E can enable institutions to use generative AI in accordance with academic or regulatory standards for suitability by integrating governance into the framework. This institutional fitness is specifically relevant since universities are increasingly being scrutinized in connection with AI applications, credential credibility, and graduate employability. HACC-E therefore does not just provide a pedagogical novelty but also a design draft of an integrated AI to be sustainable.

### 6.4. Ethical Contribution

In ethics, HACC-E adds a governance-driven, proactive attitude towards AI adoption in education. Instead of responding to ethical issues by formulating policy statements or compliance lists, the system provides ethical supervision to the learning system. By not allowing AI assessments and ensuring the human verification of the AI-assisted processes, HACC-E will be protecting academic integrity and the educator’s responsibility. The given explicit mention of ethical risks, including algorithmic bias, overtrust in AI, or data privacy issues, and the incorporation of these topics in the framework of governance are evidence of the transition to the operationalization of ethics. Such a direction has been in line with new values of responsible AI, like transparency, accountability, and human agency. Notably, HACC-E transforms ethics not as an obstacle to innovation but rather as an instrument of credible and socially appropriate educational change. Through this, the framework offers an example of an ethically based integration of AI that can be used to guide policy, institutional strategy, and future research.

### 6.5. Limitation and Future Research Direction

Despite its conceptual rigor and analytical grounding, this study has several limitations that should be acknowledged and that also define clear avenues for future research. First, the Human–AI Co-Competency Framework for Education (HACC-E) is a theoretically and analytically validated design artifact developed using a design science research approach, rather than an empirically tested instructional intervention. The framework is analytically validated through theoretical coherence, internal consistency, and alignment with established pedagogical and socio-technical principles, while also being designed to support future empirical validation through multiple research pathways. While analytical validation establishes internal coherence, theoretical alignment, and logical feasibility, it does not provide direct evidence of learning effectiveness, learner engagement, or institutional feasibility in real-world educational settings. Future studies should therefore focus on empirical validation through pilot implementations at course, program, or institutional levels, employing experimental, quasi-experimental, or mixed-methods designs to examine learning outcomes, competency attainment, and learner perceptions in AI-enhanced, mastery-based environments.

Second, the expert-based analytical evaluation employed in this study, while appropriate for early-stage design science research, was limited in its capacity to capture the diversity of disciplinary, cultural, and institutional contexts in which competency-based and AI-supported education may be implemented. Future research should expand expert validation through structured Delphi studies or large-scale stakeholder consultations involving educators, accreditation bodies, policymakers, and students, thereby strengthening the generalizability and contextual sensitivity of the framework.

Third, although HACC-E clearly articulates competency domains and aligns them with simulation-based experiential practices, the operationalization of assessment remains at a conceptual level. Subsequent research should develop and empirically test standardized competency rubrics, simulation analytics, and hybrid human–AI assessment protocols that translate framework principles into measurable and defensible evaluation instruments. Longitudinal studies would be particularly valuable for examining how mastery-based progression influences skill retention, professional adaptability, and lifelong learning behaviors over time.

Finally, while ethical governance is embedded by design in the HACC-E framework, this study does not prescribe specific institutional policies or compliance mechanisms. Future investigations should explore governance models, faculty roles, workload implications, and regulatory alignment strategies required for large-scale adoption. Collectively, these research directions will enable HACC-E to evolve from a validated conceptual framework into an empirically grounded, institutionally actionable model for responsible and competency-driven AI-enhanced education.

### 6.6. Toward Empirical Evaluation of the HACC-E Framework

While the present study adopts an analytical validation approach consistent with design science research, the rigor of the framework can be further strengthened through clearly defined pathways for empirical evaluation. Rather than constituting a finalized model, HACC-E is intended as a testable socio-technical artifact that can be examined across multiple research designs.

First, experimental and quasi-experimental studies can be employed to evaluate the impact of HACC-E on higher-order cognitive outcomes. For example, learners engaged in AI-augmented, simulation-based environments can be compared with those in traditional instructional settings, with outcome measures including problem-solving ability, critical thinking, and creative reasoning.

Second, process-oriented studies may investigate the dynamics of human–AI co-agency using interaction data. Learning analytics and trace data can be used to examine how learners engage with AI systems, the extent of cognitive offloading, and the development of metacognitive regulation over time.

Third, assessment-focused research can explore the validity and reliability of simulation-based competency evaluation. This includes examining inter-rater reliability between human evaluators and AI-supported assessment tools, as well as the alignment between observed performance and established constructs of cognitive and socio-emotional intelligence.

Fourth, qualitative and mixed-method approaches can be used to investigate socio-emotional outcomes, including learner confidence, academic anxiety, and perceptions of fairness and trust in AI-mediated environments. These dimensions are critical for understanding the broader impact of HACC-E beyond cognitive performance.

Finally, longitudinal studies may assess competency progression over time, enabling the examination of how sustained engagement with AI-augmented learning environments influences the development of adaptive expertise and transferable intelligence.

In addition to theoretical significance, the HACC-E framework offers substantial practical and research implications for educators, researchers, and educational institutions aiming to integrate generative AI into competency-based learning environments. For educators, the framework provides a systematic method for incorporating AI-assisted feedback, simulation-based experiential learning, and mastery-oriented assessment, while maintaining human oversight and pedagogical authority. It also facilitates curriculum redesign, authentic competency assessment, and the provision of higher-order cognitive, creative, and socio-emotional competencies in various educational contexts. For researchers, HACC-E establishes a robust theoretical basis for future empirical studies on human–AI co-agency, AI-supported experiential learning, learner cognition, and ethical governance in education. Additionally, the framework can serve as a reference model for developing scalable, transparent, and accountable AI-enhanced educational systems that comply with accreditation standards, quality assurance protocols, and workforce readiness requirements. By delineating these empirical and practical directions, HACC-E functions not only as a conceptual advancement but also as a foundation for systematic research and institutional adoption across diverse educational settings.

## 7. Conclusions

This paper has created and analytically confirmed the Human–AI Co-Competency Framework for Education (HACC-E) as a human and competency-based framework to unite generative artificial intelligence with modern education. The framework addresses emerging issues in the fragmentation of AI use, assessment validity, and ethical considerations of accountability by reinventing education as a socio-technical system in which competencies, AI-enhanced learning processes, simulation-based experiential practice, and governance are interrelated. The research, based on a design science research design, has proven that HACC-E has attained pedagogical coherence, facilitated authentic and assessable competency evidence, and met the demands of outcome-based accreditation, as well as integrating ethical oversight by design as opposed to an addendum.

The key value that this work brings is that educational theory and practice are no longer based on the integrated aspects of AI tools. HACC-E can maintain educator control and capitalize on the opportunity offered by AI to provide personalized and formative support by operationalizing human and AI co-agency explicitly. The framework also addresses longstanding issues surrounding competency-based education by ensuring that simulation-based learning is the primary mode of performance-based evaluation and by implementing processes that enhance the validity and transparency of learning outcomes. Notably, this research does not claim that it is empirically effective; instead, it lays out a rigorously grounded conceptual artifact and offers a basis for systematic application and testing.

The empirical study of the HACC-E framework provided through course-level and program-level application in different contexts must be considered in the future. Experimental and quasi-experimental studies on potential learning outcomes, learner engagement, and the transfer of skills in AI-enhanced, competency-driven contexts may follow. Other competencies that can be further developed to enhance and validate competency rubrics, simulation analytics, and hybrid human–AI assessment protocols will be required within the framework. Longitudinal research would be able to examine how mastery-based advances would impact lifetime learning, professional flexibility, and moral reasoning as time goes by. At the institutional level, the research that should be conducted in the future is an analysis of models of governance, roles of faculty, and policy implications related to the scaling up of HACC-E in the domain of accreditation and the regulatory environment. When taken together, these research directions will give rise to the responsible, evidence-based adoption of generative AI in the education sector, so that technological innovation, instead of deteriorating outcomes, will contribute to human learning and judgment.

## Figures and Tables

**Table 1:** Comparison of traditional education model and the HACC-E framework.

Dimension	Traditional Education Model	HACC-E Framework
Curriculum structure	Course-centric	Competency-driven
Learning progression	Time-based	Mastery-based
Assessment focus	Exams and grades	Performance evidence
Role of technology	Supplementary	Integral and systemic
Faculty role	Content delivery	Mentoring, validation, governance
Student role	Passive recipient	Active co-constructor of competence
Evidence of learning	Indirect	Direct and observable

**Table 2:** Theoretical integration underpinning the HACC-E framework.

Theoretical Component	Core Principle	Role in HACC-E	Relationship to Other Components
Constructivism	Knowledge is actively constructed through experience and interaction	Informs learner-centered, experiential, and reflective learning processes	Provides the pedagogical foundation for simulation-based learning and competency development
Socio-Technical Systems Theory	Human and technological elements form interdependent systems requiring joint optimization	Guides the integration of AI within educational environments while maintaining human control	Provides the structural basis for human–AI co-agency and governance mechanisms
Human–AI Co-Agency	Agency is distributed between humans and AI with defined roles and accountability	Defines interaction model between learners, educators, and AI systems	Operationalizes socio-technical principles within learning processes and supports constructivist engagement
Competency-Based Education (CBE)	Learning is measured through demonstrated mastery of competencies	Structures learning outcomes, progression, and assessment criteria	Aligns with constructivist learning and is enabled by AI-supported feedback and simulation-based assessment
Simulation-Based Learning	Learning occurs through contextualized, scenario-based experiences	Translates competencies into observable performance in realistic environments	Supports constructivist principles and enables the enactment of co-agency in practice

**Table 3:** Core educational competency domains.

Domain	Description
Conceptual understanding	Mastery of disciplinary and interdisciplinary knowledge
Problem formulation	Ability to define and structure complex problems
Systems & critical thinking	Understanding interdependencies and consequences
Creativity & innovation	Generation of novel and effective solutions
Digital & AI literacy	Effective and ethical use of digital technologies
Ethics & sustainability	Responsible and value-based decision-making
Communication & co-agency	Interpersonal and interdisciplinary engagement
Lifelong learning	Continuous self-directed skill development

**Table 4:** Illustrative Sub-Indicators and Pedagogical Scenarios for Core Competencies.

Competency Domain	Sub-Indicators	Example Pedagogical Scenario
Problem formulation	Ability to define ill-structured problems; identification of constraints and variables	In a research writing task, learners use AI-assisted literature exploration to refine a research question, identify gaps, and justify methodological choices
Systems & critical thinking	Analysis of interdependencies; evaluation of alternative solutions; evidence-based reasoning	In a policy simulation, learners analyze multi-variable scenarios (e.g., economic, environmental trade-offs) and justify decisions using AI-supported data interpretation
Creativity & innovation	Generation of novel ideas; iterative refinement; synthesis of diverse inputs	In a design-thinking simulation, learners co-create solutions with AI by generating multiple prototypes and critically selecting viable innovations
Digital & AI literacy	Critical evaluation of AI outputs; awareness of bias and limitations; ethical use	In an AI-assisted assignment, learners compare AI-generated responses with domain knowledge, identify inaccuracies, and justify acceptance or rejection
Communication & collaboration	Articulation of ideas; interdisciplinary coordination; feedback integration	In a collaborative simulation, learners use AI to draft reports, then refine them through peer critique and instructor validation
Ethics & sustainability	Ethical reasoning; evaluation of societal impact; responsible decision-making	In a simulated case study, learners assess ethical implications of AI-driven decisions (e.g., bias in automated hiring systems) and propose mitigation strategies

**Table 5:** Ethical risks and mitigation strategies.

Risk	Description	Mitigation Strategy
Algorithmic bias	Skewed or inequitable AI outputs	Human validation and auditing
Overreliance on AI	Reduced critical engagement	AI literacy and reflective tasks
Assessment validity	Automated evaluation errors	Hybrid human–AI assessment
Data privacy	Misuse of learner data	Institutional data governance
Academic integrity	Misrepresentation of AI-generated work	Clear policies and oversight

## Data Availability

The data that support the findings of this study are available from the corresponding authors upon reasonable request.
